# Rift Valley Fever Virus Propagates in Human Villous Trophoblast Cell Lines and Induces Cytokine mRNA Responses Known to Provoke Miscarriage

**DOI:** 10.3390/v13112265

**Published:** 2021-11-12

**Authors:** Yong-Dae Gwon, Seyed Alireza Nematollahi Mahani, Ivan Nagaev, Lucia Mincheva-Nilsson, Magnus Evander

**Affiliations:** Department of Clinical Microbiology, Umeå University, 901 85 Umeå, Sweden

**Keywords:** rift valley fever virus, human villous trophoblast, cytokine, interferon, inflammatory cytokines, miscarriage

## Abstract

The mosquito-borne Rift Valley fever (RVF) is a prioritised disease that has been listed by the World Health Organization for urgent research and development of counteraction. Rift Valley fever virus (RVFV) can cause a cytopathogenic effect in the infected cell and induce hyperimmune responses that contribute to pathogenesis. In livestock, the consequences of RVFV infection vary from mild symptoms to abortion. In humans, 1–3% of patients with RVFV infection develop severe disease, manifested as, for example, haemorrhagic fever, encephalitis or blindness. RVFV infection has also been associated with miscarriage in humans. During pregnancy, there should be a balance between pro-inflammatory and anti-inflammatory mediators to create a protective environment for the placenta and foetus. Many viruses are capable of penetrating that protective environment and infecting the foetal–maternal unit, possibly via the trophoblasts in the placenta, with potentially severe consequences. Whether it is the viral infection per se, the immune response, or both that contribute to the pathogenesis of miscarriage remains unknown. To investigate how RVFV could contribute to pathogenesis during pregnancy, we infected two human trophoblast cell lines, A3 and Jar, representing normal and transformed human villous trophoblasts, respectively. They were infected with two RVFV variants (wild-type RVFV and RVFV with a deleted NSs protein), and the infection kinetics and 15 different cytokines were analysed. The trophoblast cell lines were infected by both RVFV variants and infection caused upregulation of messenger RNA (mRNA) expression for interferon (IFN) types I–III and inflammatory cytokines, combined with cell line-specific mRNA expression of transforming growth factor (TGF)-β1 and interleukin (IL)-10. When comparing the two RVFV variants, we found that infection with RVFV lacking NSs function caused a hyper-IFN response and inflammatory response, while the wild-type RVFV suppressed the IFN I and inflammatory response. The induction of certain cytokines by RVFV infection could potentially lead to teratogenic effects that disrupt foetal and placental developmental pathways, leading to birth defects and other pregnancy complications, such as miscarriage.

## 1. Introduction

Rift Valley fever (RVF) is one of the prioritised diseases that since 2018 has been listed by the World Health Organization for urgent research and development of counteraction [1]. RVF is a zoonotic disease, caused by Rift Valley fever virus (RVFV), a negative-strand RNA virus of the family *Phenuiviridae* (former family *Bunyaviridae*), genus *Phlebovirus*, present in Africa and the Arabian Peninsula [2,3]. RVFV is transmitted to livestock by mosquitoes, predominantly by species of the genera *Aedes* and *Culex*, and RVFV can infect and cause disease in both livestock and humans [4]. RVFV can potentially cause disease directly by a cytopathogenic effect (CPE) in the infected cell/organ and/or indirectly by inducing immune responses that contribute to the pathogenesis. The consequence of RVFV infection in livestock varies from mild symptoms to abortion or foetal malformation [5]. Sheep especially show high susceptibility to RVFV infection, with abortion in up to 90% of infected pregnant ewes, and the mortality in newborn lambs is almost 100% [6,7,8]. In many cases, RVFV was recovered from both aborted livestock foetal material and placental tissue [9]. Pregnant sheep inoculated with live attenuated vaccine strains, lacking a functional non-structure protein S (NSs), showed teratogenic effects similar to wild-type virus, and either miscarried or newborn lambs had malformations [10,11,12].

In humans, RVFV infection is in most cases a self-limiting febrile illness. However, 1–3% of cases develop more severe symptoms of haemorrhagic fever, neurological disorders or blindness, which could be lethal [7,13]. Mosquitoes transmit RVFV to humans, but direct contact with infected animals, aborted foetal material, and consumption of raw milk also have been shown to correlate with transmission in endemic RVFV regions [14,15,16]. As in livestock, RVFV, transmitted to pregnant women, has been associated with miscarriage and foetal disease [17]. Studies of RVFV vertical transmission in humans and its effect on human pregnancy and the foetus have so far been scarce and mostly limited to case reports, such as an RVFV-infected pregnant woman delivering a newborn with a rash, an enlarged liver and spleen, and jaundice [18]. In another case, a pregnant woman showed clinical symptoms of RVFV a few days before giving birth and delivered an infant who subsequently died of RVFV within a week [19]. Furthermore, there was a strong association between late-term miscarriage and confirmed RVFV infection in pregnant women (odds ratio 7.4) in a cross-sectional study from Sudan [13]. Together, these reports suggest that RVFV infection during pregnancy could be associated with miscarriages.

From an immunologic point of view, pregnancy is a finely tuned condition where pro-inflammatory and anti-inflammatory mediators harmonise, creating a protective milieu for the foetal–maternal unit [20]. Inheriting 50% of its genes from the father, the foetal semi-allograft and its placenta are not recognised as a transplant, but accepted and allowed to grow and develop in the uterine cavity. Several immune cells and mechanisms, such as cytokine responses in the local and systemic microenvironment, downregulation of cytotoxicity, and a bias towards humoral immunity, are involved in the adaptation of the maternal immune system towards foetal immune tolerance [21,22,23,24]. The general T helper (Th) 2 deviation of the cytokine profile downregulates the cytotoxic response and suppresses adaptive immunity, making pregnant women generally vulnerable to viral infections. Upregulation of the innate immune responses partly compensates for the constitutive impairment of the cytotoxic adaptive immune responses, but it is not enough to reverse the fatal outcome of certain viral and parasitic infections that are vertically transmitted during pregnancy. The placenta is a key organ for pregnancy success. The function of the human haemochorial placenta is associated exclusively with villous trophoblasts. These cells are important for hormonal regulation during pregnancy, providing nutritional and oxygen support to the foetus, and secretion of various cytokines and immunoregulatory factors, which can alter the maternal immune responses in healthy or pathological pregnancy [24,25]. Many viruses with known vertical transmission infect the villous trophoblasts of the placenta; the best known is rubella virus [26]. In addition, cytomegalovirus, dengue virus, and HIV also cause vertical infections that could lead to teratogenic effects, while other viruses such as parvo B19 virus, BK polyomavirus, human papillomavirus, herpes simplex virus type-1, herpes simplex virus type-2, and hepatitis B virus have been associated with miscarriages [26,27]. To infect the foetus, a virus could take either a placental or paraplacental route of transmission. For example, Zika virus spreads from the basal and parietal decidua to chorionic villi and amniochorionic membranes [28]. RVFV has been shown to target cyto- and syncytiotrophoblasts [12,29], and based on known transmission routes, we hypothesise that RVFV first infects the villous syncytiotrophoblasts of the placenta that are in direct contact with the maternal blood and thereafter reach the foetal circulation in the chorionic villi, thus producing foetal haematogenic RVFV transmission. Once in contact with the foetus, the virus can infect many different tissues and organs. Whether it is the viral infection per se, the immune response, or both that contribute to the pathogenesis of miscarriage remains unknown. In general, the individual host innate response to RVFV infection, specifically inflammation, is likely an important contributor to pathogenesis. Interestingly, dysregulation of the inflammatory response during RVFV infection has been shown [30]. Thus, it is crucial to understand the cytokine profiles upon infection of RVFV in the infected trophoblasts.

Cytokines coordinate and determine the subsequent immune response and provide various signals that regulate important biological events such as growth, differentiation, inflammation, cytotoxicity, and immune suppression. Different cytokine messenger RNA (mRNA) profiles, designated Th1, Th2, Th3/Tr1, and Th17, comprising a set of synergistically acting cytokines, are associated with the promotion of different immune responses. Thus, a cytokine profile dominated by interferon (IFN)-γ, interleukin (IL)-12, and IL-15 (Th1) promotes cytotoxicity; a cytokine profile dominated by IL-4, IL-5, and IL-13 (Th2) promotes humoral immunity; a cytokine profile of IL-1β, IL-6, IL-8, IL-17, tumour necrosis factor (TNF)-α, and TNF-β/lymphotoxin-alpha (LTA) promotes inflammation; and a cytokine profile of transforming growth factor (TGF)-β1 and IL-10 (Th3/Tr1) promotes immunosuppression as well as innate and adaptive T regulatory cell (Treg) development [31].

In a mouse model, infection with the candidate RVFV vaccine strains (MP12 and clone 13, which have either deletions or mutations in the NSs-gene) showed increased secretion of T helper (Th)1-associated antiviral cytokines, chemokines, and various interleukins, whereas infection with wild-type RVFV (with a functional NSs-gene) almost entirely ablated the immune response, because NSs inhibits the host antiviral response [32]. In addition, the candidate RVFV vaccine strains have been shown to have teratogenic effects in sheep; however, the mechanism and the possible effect on humans have not been elucidated [11,33].

wt RVFV can inhibit a pro-inflammatory response in infected human monocyte-derived macrophages while NSs-deleted recombinant RVFV showed no inhibition [34]. wt RVFV also upregulates TNF-α in human primary small airway lung epithelial cells [35]. Moreover, cytokine profiling in mouse bone-marrow-derived macrophages after infection with wt RVFV, or attenuated strains has been described [32]. Together, these findings highlight the importance of the role of the RVFV NSs protein for regulating the host response to RVFV infection in different species and cell types. However, the association between the cellular host response and RVFV infection in human trophoblasts is unclear.

In this study, we characterised the infection capabilities and cytokine response in two different human trophoblast cell lines (A3 and Jar) by infecting them with different RVFV strains, either the wild-type (wt) strain or NSs-deleted recombinant RVFV.

## 2. Materials and Methods

### 2.1. Cells

Two human trophoblast cell lines, A3 and Jar, were used. A3 comprises immortalised trophoblasts derived from a normal human pregnancy, kindly provided by Prof. Gil Mor (Wayne State University, Detroit, MI, USA) [36]. The choriocarcinoma cell line Jar was purchased from ATCC. For maintenance, both cell lines were grown in RPMI 1640 medium (Thermo Scientific, Waltham, MA, USA) supplemented with 10% foetal bovine serum (FBS, Gibco, NY, USA), 10 mM HEPES (4-[2-hydroxyethyl]- 1-piperazineethanesulfonic acid, Thermo Scientific), 1 mM sodium pyruvate (Thermo Scientific), 2% MEM amino acids (Thermo Scientific), 1% MEM non-essential amino acids (Thermo Scientific), and 0.2% penicillin/streptomycin (PE/ST, Thermo Scientific) at 37 °C. For the infection experiment, RPMI was supplemented with 1% FBS.

### 2.2. RVFV Strains

**Figure 1:**
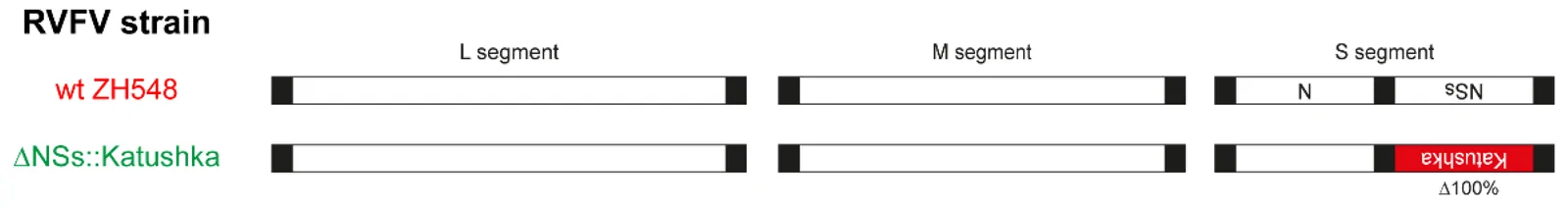
Schematics of the Rift Valley fever virus (RVFV) strains used in this study. The image shows the genomes of two different RVFV strains. The ΔNSs::Katushka strain was derived from the wild-type (wt) ZH548 strain by replacement of the NSs gene with the far-red fluorescent protein Katushka gene.

Two RVFV strains were selected for this study: the wt ZH548 strain and a recombinant RVFV virus derived from the ZH548 strain by substituting the NSs protein with the far-red fluorescent protein Katushka, hereafter termed ΔNSs::Katushka [37]. The viral genomic organisation of both strains is presented schematically in Figure 1.

For viral propagation, Vero B4 cells [38] were grown in Dulbecco’s Modified Eagle’s Medium (DMEM) (Thermo Scientific) supplemented with 5% FBS and 0.2% PE/ST. One day before viral inoculation, 2 × 10^6^ Vero B4 cells were seeded in a T75 flask (Sarstedt, Nümbrecht, Germany). Next, cells were infected with RVFV at a multiplicity of infection (MOI) of 0.01 for 1 h and maintained in DMEM with a lower FBS concentration (1%). The supernatant containing virions was collected 72 h post-infection and titrated by using a plaque assay. All work involving the wt ZH548 strain was performed under biosafety laboratory 3 (BSL-3) conditions; the ΔNSs::Katushka strain used in the study was handled in BSL-2 conditions, as permitted by the Swedish Work Environment Authority.

### 2.3. Measurement of RVFV Gn Protein Expression

A day before infection, 2 × 10^5^ A3 or Jar cells/well were seeded in 12-well plates (Nunc, Roskilde, Denmark). On the day of infection, the growth medium was removed and cells were infected with two different viruses at an MOI of 1 for 1 h at 37 °C. Then, virus inoculum was removed, fresh RPMI with 1% FBS was added, and the incubation continued for 23 h at 37 °C in 5% CO_2_.

After 24 h of infection, the cells were fixed with 4% paraformaldehyde and washed with phosphate-buffered saline (PBS). The fixed cells were stained with mouse anti-RVFV Gn protein antibody (015A-03444, European virus archive) diluted in 2% bovine-serum albumin (BSA) in PBS [39]. Incubation with primary antibodies was followed by three washes and incubation with secondary anti-mouse antibody conjugated to Alexa Fluor 488 (Thermo Scientific). Nuclei were counterstained with 300 nM 4′,6-diamidino-2-phenylindole (DAPI) in PBS.

Cytation 5 Cell Imaging Multi-Mode Reader (BioTek, Winooski, VT, USA) identified green fluorescent protein (GFP)- or DAPI-expressing cells and quantified the fluorescence intensity in each well.

### 2.4. Total Cellular RNA Extraction and Real-Time Quantitative Cytokine mRNA Expression Analysis

To perform the cytokine mRNA expression analysis, 2 × 10^5^ A3 or Jar cells were infected at an MOI of 1; infected cells were harvested for RNA isolation at 0, 6, and 24 h post-infection (hpi). For total cellular RNA isolation, the cells were washed with PBS and RNA was extracted by using the RNeasy^®^ Mini kit (QIAGEN, Hilden, Germany) according to the manufacturer’s instructions. RNA yield and purity were assessed by spectrophotometry (NanoDrop, Thermo Scientific).

Reverse transcription–quantitative polymerase chain reaction (RT-qPCR) was performed on an ABI StepOnePlus™ Real-Time PCR system. Fifty nanograms of total RNA/well in a 20 μL reaction volume was used in all tests and run for 40 cycles with factory default settings of qPCRBIO Probe 1-step Go Hi-ROX kit and TaqMan^®^ FAM/MGB probe assays (all purchased from Thermo Scientific). mRNA expression was measured for the following cytokines: IFNα1, IFNβ1, IFNγ, IFNλ, IL-4, IL-5, IL-1β, IL-6, IL-8, TNF-α, IL-10, and TGF-β1, chosen to discriminate between cytotoxic (Th1), humoral (Th2), and inflammatory and immunosuppressive/Treg (Th3/Tr1) immune responses. In addition, markers for apoptosis, autophagy, and cell survival were assessed by mRNA transcription of TP53, MAPLC3A, and NF-κB1, respectively. Their assay ID are as follows: IFNα1 (Hs00256882_s1), IFNβ1 (Hs01077958_s1), IFNγ (Hs00989291_m1), IFNλ (Hs00601677_g1), IL-4 (Hs00174122_m1), IL-5 (Hs00174200_m1), IL-1β (Hs01555410_m1), IL-6 (Hs00985639_m1), IL-8 (Hs00174103_m1), TNF-α (Hs00174128_m1), IL-10 (Hs00961622_m1), TGF-β1 (Hs99999918_m1), TP53 (Hs01034249_m1), MAP1LC3A (Hs01076567_g1), and NF-κB1 (Hs00765730_m1).

Ct values for the endogenous housekeeping gene 18S rRNA were measured by using the Eukaryotic 18S rRNA Endogenous Control VIC/MGB Assay (Cat No. 4319413E, Thermo Scientific) (Figure S1). Cytokine mRNA amplification of PMA/ionomycin-stimulated peripheral blood mononuclear cells (PBMCs) from healthy donors was used as a positive control for the cytokine mRNA assays; template omission was used as a negative control. Tests were analysed with the StepOne Software 2.3. The raw data were evaluated with the ΔΔCt method, resulting in relative quantities (RQ) (Table S1). For each cell line, the RQ for each different condition (viruses or time) was compared with the baseline (mock-infected cells at time point 0), producing a fold difference.

### 2.5. Statistical Analysis

Means and standard deviations (SD) were calculated with GraphPad Prism 7.0 software. All statistical analyses were performed by using either multiple t-tests or one-way analysis of variance (ANOVA) plus Dunnett’s post hoc analysis by GraphPad Prism 7.0 software; *p* < 0.05 was considered statistically significant.

## 3. Results

### 3.1. Experimental Setup

The great majority of viral intrauterine infections in mammalian pregnancy are transmitted through the placenta. The human hemochorial placenta is unique in its anatomical and functional organization and thus there are no suitable animal models for in vivo studies that reflect the human placenta, that could replace in vitro studies with cell lines, organoids and explant cultures. The placental chorionic villi are in direct contact with the maternal blood, and are covered with villous trophoblast cells that comprise more than 80% of all placental cells. Because the villous trophoblast is the principal placental cell type, which secretes a huge variety of proteins and signal substances regulating and ensuring pregnancy success, we focused our studies on two trophoblast cell lines, A3 and Jar, representing normal and transformed human villous trophoblast respectively.

Both wt and NSs-deleted recombinant RVFV strains were used for infection of the trophoblast cell lines. The wt RVFV is capable of escaping cytotoxic response through manipulation of the host response by the RVFV’s NSs non-structural protein from the S gene (NSs protein), functioning as an IFN antagonist [40]. We included a NSs-deleted recombinant RVFV strain to evaluate the effect on the cytokine mRNA transcription compared to the effect of wt RVFV. We aimed at determining whether the NSs-deleted strain, similar to the wt, evoked cytokine responses in the villous trophoblast that could be dangerous to the pregnancy and fetal development, or induced cytokines known to be involved in inducing miscarriage.

### 3.2. RVFV Replicates in Immortalised Human Normal Trophoblast and Human Choriocarcinoma Cell Lines

**Figure 2:**
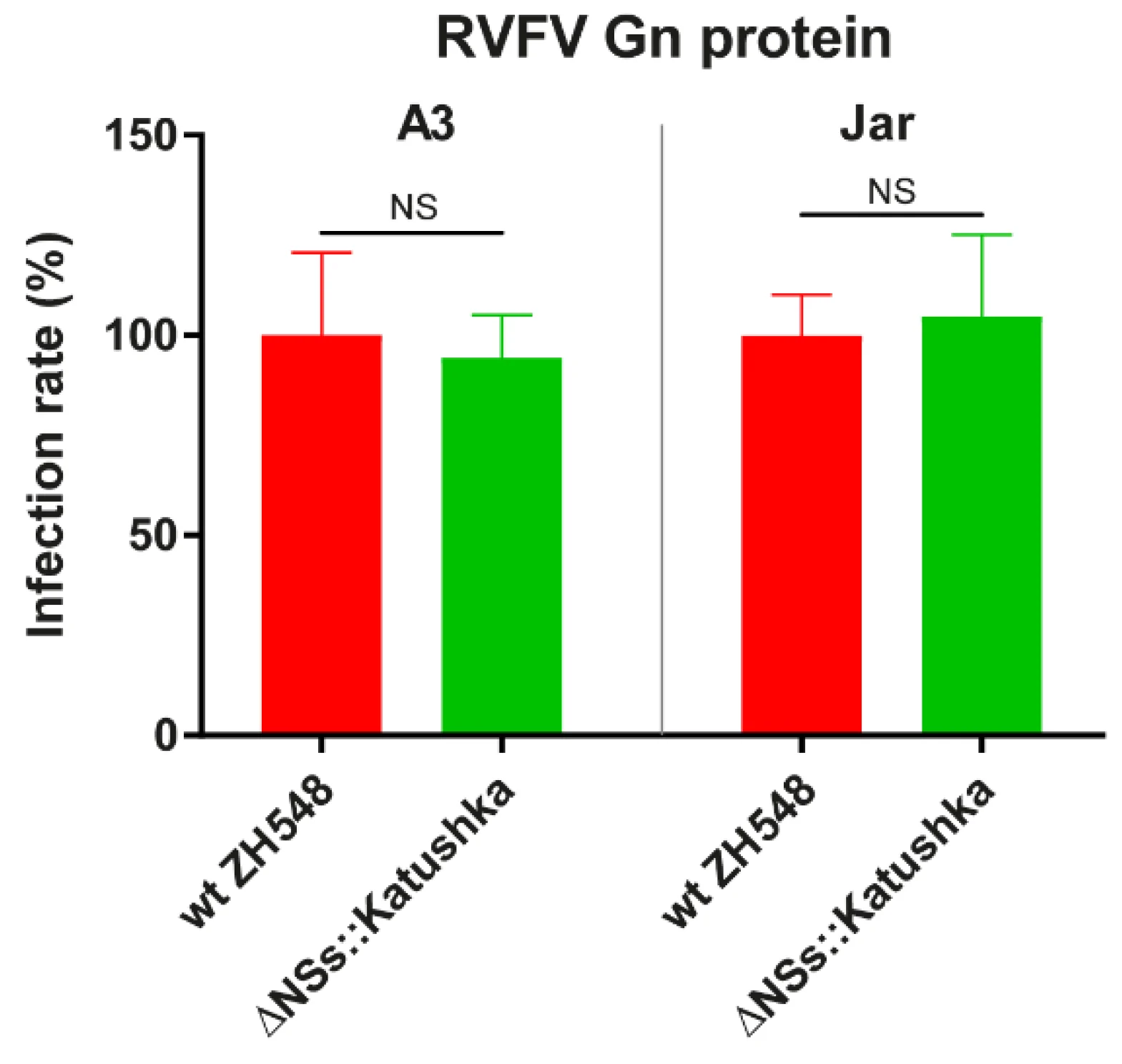
Rift Valley fever virus (RVFV) Gn protein expression in the A3 and Jar cell lines. The cell lines were infected at a multiplicity of infection of 1 and the Gn protein was detected by using an anti-RVFV Gn monoclonal antibody, and a secondary anti-mouse antibody conjugated to Alexa Fluor 488. Cytation 5 Cell Imaging Multi-Mode Reader identified green fluorescent protein (GFP)- or 4′,6-diamidino-2-phenylindole (DAPI)-expressing cells and quantified the fluorescence intensity in each well. Experiments were performed in triplicate and repeated twice. The bar plus error bar indicates the mean ± standard deviation. Statistical significance was determined by multiple t-test. *p* values are indicated (NS = not significant).

To investigate whether the two trophoblast cell lines were permissive to RVFV infection, we infected them with two RVFV strains at an MOI of 1 and measured the RVFV Gn protein expression at 24 hpi. The trophoblast cell lines were permissive to infection with both RVFV strains and showed a similar infection rate with no significant differences (Figure 2). Thus, we decided to use the same MOI for cytokine profiling analysis. Infection with the RVFV variants resulted in a CPE in A3 and Jar cells. This CPE was initiated at 24 hpi and reached approximately 90% at 72 hpi (data not shown).

### 3.3. Interferon Expression in RVFV-Infected Human Trophoblast Cell Lines

We infected human trophoblast cell lines with wt ZH548 or ΔNSs::Katushka to examine the expression of IFN types I (IFNα1 and β1), II (IFNγ) and III (IFNλ).

**Figure 3:**
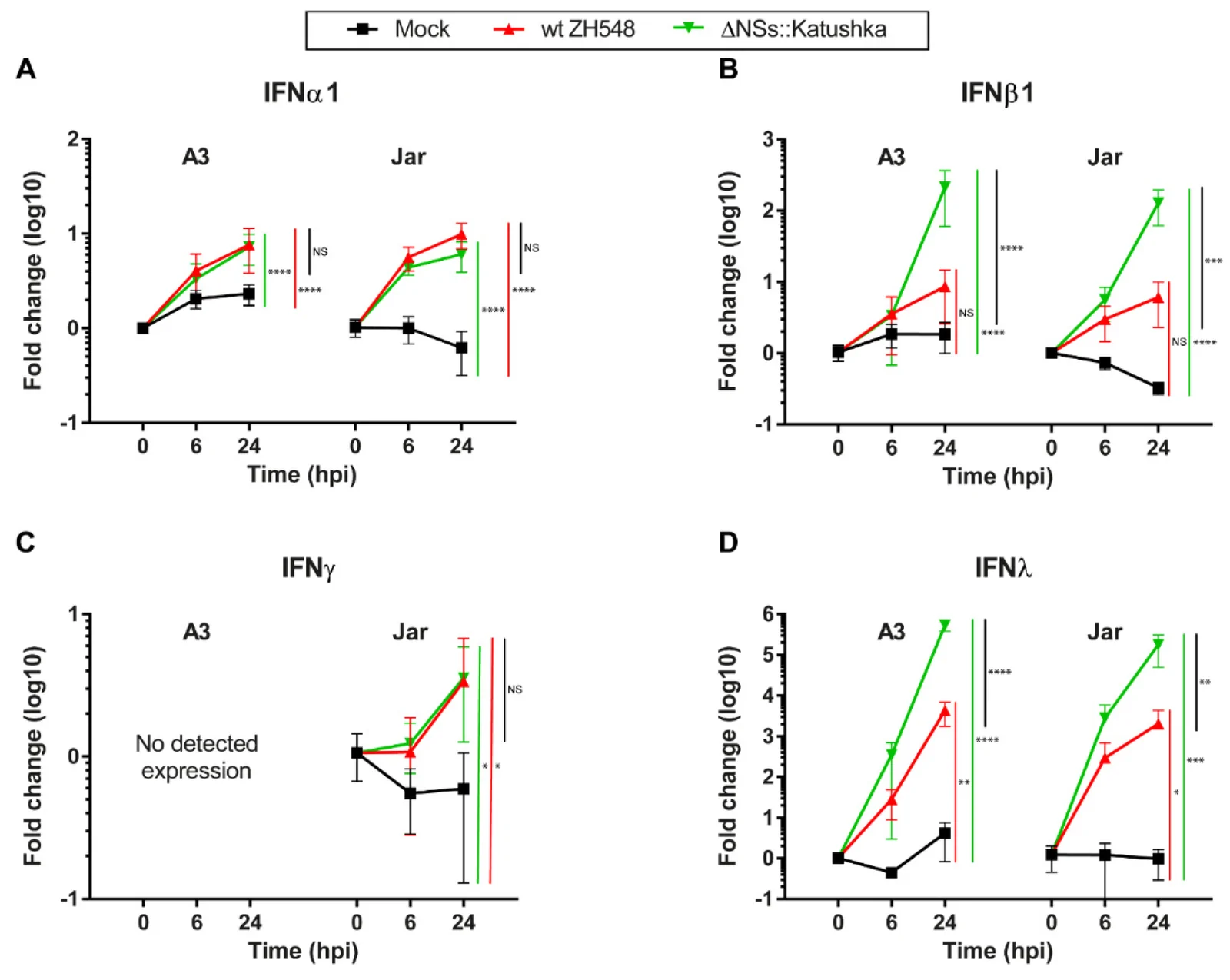
Interferon (IFN) mRNA response in A3 (immortalised human normal trophoblasts) and Jar (human choriocarcinoma) cell lines infected with two different Rift Valley fever virus (RVFV) strains. The cell lines were infected at a multiplicity of infection of 1 and cell lysates were harvested at 0 h and at 6 or 24 h post-infection (hpi). Total cellular RNA was extracted from harvested cell lysate and used for detection of IFNα1 (**A**), IFNβ1 (**B**), IFNγ (**C**), and IFNλ (**D**) RNA using reverse transcription-quantitative polymerase chain reaction (RT-qPCR) with TaqMan^®^ FAM/MGB probe assays. Experiments were performed in triplicate and repeated twice. The symbol plus error bar indicates the mean ± standard deviation. Statistical significance was determined by one-way analysis of variance (ANOVA) plus Dunnett’s post hoc analysis. The data from 24 hpi was used for multiple group comparisons and the significance of *p* values is indicated by the asterisks; red line = mock vs. wt ZH548; green line = mock vs. ΔNSs::Katushka; black line = wt ZH548 vs. ΔNSs::Katushka (* *p* < 0.05, ** *p* < 0.01, *** *p* < 0.001, **** *p* < 0.0001, NS = not significant).

The type I IFN transcription was elevated in all RVFV-infected cells at 24 hpi, but with higher IFNβ1 and IFNλ expression in cells infected with ΔNSs::Katushka. For IFNα1, the expression levels were increased 6–10-fold, with no discernible differences between the wt and ΔNSs::Katushka RVFV (Figure 3A). For IFNβ1 transcription, a significant increase was detected in cells infected with ΔNSs::Katushka RVFV compared with wt RVFV. The IFNβ1 mRNA increment in ΔNSs::Katushka RVFV-infected cells at 24 hpi was 212-fold for A3 cells and 128-fold for Jar cells, while the wt RVFV-infected cells only displayed a 6–8-fold increase at 24 hpi (Figure 3B).

To determine the IFN type II response, we evaluated the level of the Th1 immune-response associated IFNγ mRNA transcription after RVFV infection. We observed a low-level increase in IFNγ mRNA transcription in RVFV-infected Jar cells at 24 hpi, but it was not the same extent as IFN type I response. In the A3 cells, originating from normal tissue, no IFNγ mRNA expression was detected (Figure 3C).

We further examined IFNλ (IFN type III) mRNA transcription and observed that the IFNλ mRNA response against infection with both RVFV strains had the highest relative increase for all three types of IFN responses investigated. In both A3 and Jar cells, the IFNλ elevation was high—the fold change was between 2.0 × 10^3^- and 5.3 × 10^5^-fold at 24 hpi. The infection with ΔNSs::Katushka RVFV showed significantly higher IFNλ expression than wt RVFV-infected cells, although the fold induction of IFNλ in wt RVFV-infected cells was still at a high level (Figure 3D).

From the results, we concluded that RVFV infection triggered type I and type III IFN response in human trophoblast cell lines, with strongly induced expression of IFNβ1 and IFNλ, especially when cells were infected with the NSs-deleted RVFV. The lack of expression of the Th1 immune response–associated IFNγ in normal trophoblast cell line (A3) is expected and can be seen as a control for the performed IFN mRNA analyses. By contrast, the choriocarcinoma cell line JAR had some aberrant, albeit low, IFNγ mRNA expression.

### 3.4. Th2 Cytokine mRNA Response in Human Trophoblast Cell Lines after RVFV Infection

**Figure 4:**
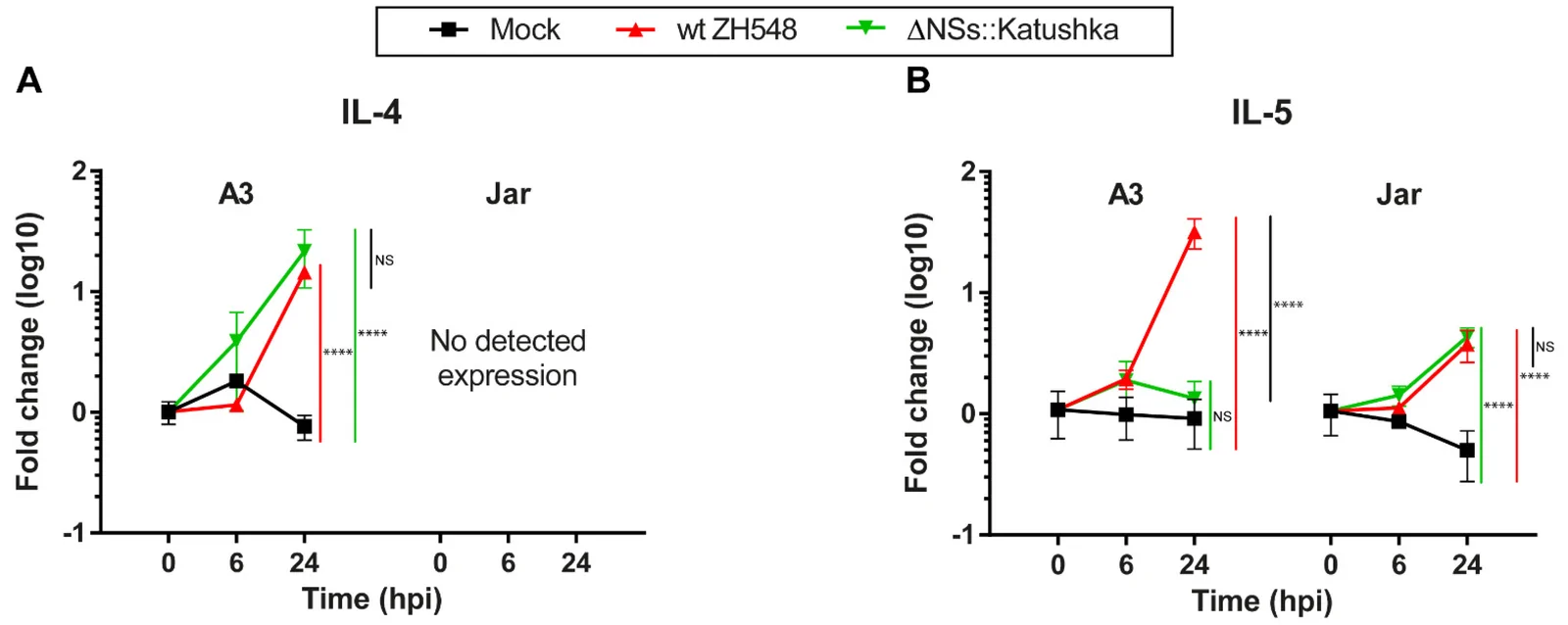
Th2 cytokine mRNA response in A3 cells (immortalised human normal trophoblast) and Jar (human choriocarcinoma) cell lines infected with two different Rift Valley fever virus (RVFV) strains. Both cell lines were infected at a multiplicity of infection of 1 and cell lysates were harvested at 6 or 24 h post-infection (hpi). The total cellular RNA was extracted from harvested cell lysates and used for detection of interleukin (IL)-4 (**A**) and IL-5 (**B**) RNA by using reverse transcription-quantitative polymerase chain reaction (RT-qPCR) with TaqMan^®^ FAM/MGB probe assays. Experiments were performed in triplicate and repeated two times with similar results. The symbol plus error bar indicates the mean ± standard deviation. Statistical significance was determined by one-way analysis of variance (ANOVA) plus Dunnett’s post hoc analysis. The data from 24 hpi was used for multiple group comparison and the significance of *p* values is indicated by the asterisks; red line = mock vs. wt ZH548; green line = mock vs. ΔNSs::Katushka; black line = wt ZH548 vs. ΔNSs::Katushka (**** *p* < 0.0001, NS = not significant).

Next, we evaluated the expression of the Th2 cytokines IL-4 and IL-5 after RVFV infection. In A3 cells at 24 hpi, the RVFV variants showed a 14–21-fold upregulation of IL-4 transcription. In Jar cells, expression of IL-4 RNA was not detectable (Figure 4A).

At 24 hpi, A3 cells infected with wt RVFV showed a 31-fold increase in IL-5 RNA expression and Jar cells showed a 3.7-fold increase, whereas ΔNSs::Katushka RVFV-infected cells showed a similar increase only in Jar cells (Figure 4B). The data showed that RVFV induced only moderately increased expression of Th2 cytokine mRNAs in the human trophoblast cell lines.

### 3.5. The Inflammatory Cytokine mRNA Response in Human Trophoblast Cell Lines after RVFV Infection

To investigate the inflammatory response following RVFV infection of human trophoblast cell lines, we examined the mRNA expression of IL-1β, IL-6, IL-8, and TNF-α.

**Figure 5:**
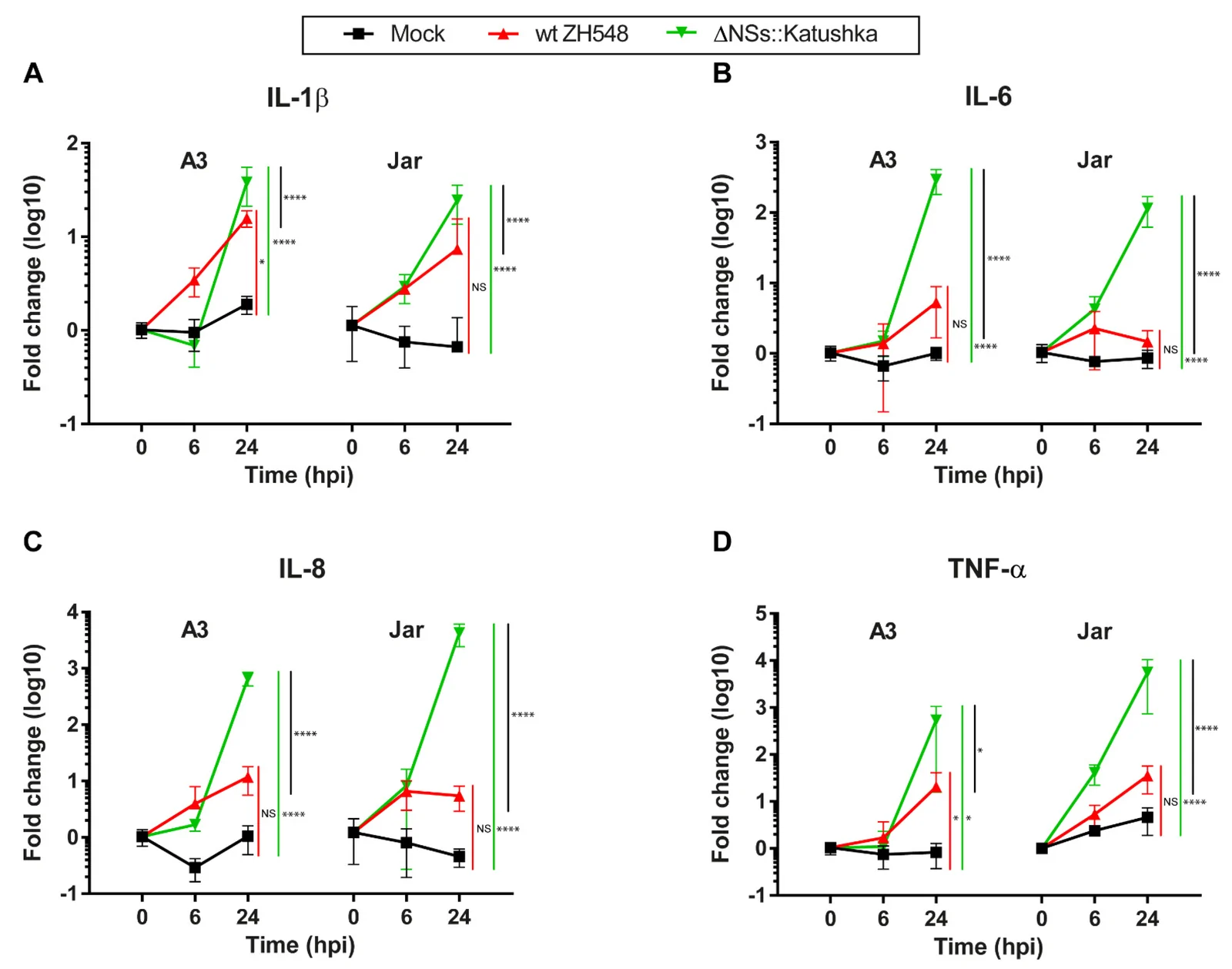
The inflammatory cytokine response in A3 (immortalised human normal trophoblast) and Jar (human choriocarcinoma) cell lines infected with two different Rift Valley fever virus (RVFV) strains. Both cell lines were infected at a multiplicity of infection of 1 and cell lysates were harvested at 6 or 24 h post-infection (hpi). The total cellular RNA was extracted from harvested cell lysates and used for detection of the inflammation-associated cytokine messenger RNA (mRNA) (interleukin (IL)-1β (**A**), IL-6 (**B**), IL-8 (**C**), and tumour necrosis factor (TNF)-α (**D**)) by using reverse transcription-quantitative polymerase chain reaction (RT-qPCR) with TaqMan^®^ FAM/MGB probe assays. Experiments were performed in triplicate and repeated twice. The symbol plus error bar indicates the mean ± standard deviation. Statistical significance was determined by one-way analysis of variance (ANOVA) plus Dunnett’s post hoc analysis. The data from 24 hpi was used for multiple group comparison the significance of *p* values is indicated by the asterisks; red line = mock vs. wt ZH548; green line = mock vs. ΔNSs::Katushka; black line = wt ZH548 vs. ΔNSs::Katushka (* *p* < 0.05, **** *p* < 0.0001, NS = not significant).

In general, the mRNA expression of all four inflammatory cytokines was elevated, and infection with ΔNSs::Katushka RVFV resulted in higher expression of inflammatory cytokine mRNAs compared with infection with wt RVFV. For IL-6, IL8, and TNF-α, the ΔNSs::Katushka RVFV-infected cells showed a > 10^2^- to > 10^3^-fold increase in A3 cells and a > 10^3^-fold increase in Jar cells, whereas wt RVFV-infected A3 and Jar cells showed a 1–35-fold increase in these cytokines (Figure 5B–D). This upregulation of all four inflammatory cytokines in ΔNSs::Katushka RVFV-infected cells was significantly higher than the wt RVFV-infected cells (Figure 5).

Overall, these results suggested that RVFV infection (wt ZH548 and ΔNSs::Katushka) induced an inflammatory cytokine response in human trophoblast cell lines. However, ΔNSs::Katushka RVFV was a more powerful inducer of an inflammatory cytokine response.

### 3.6. Immunosuppressive/Treg Cytokine mRNA Response in Human Trophoblast Cell Lines after RVFV Infection

**Figure 6:**
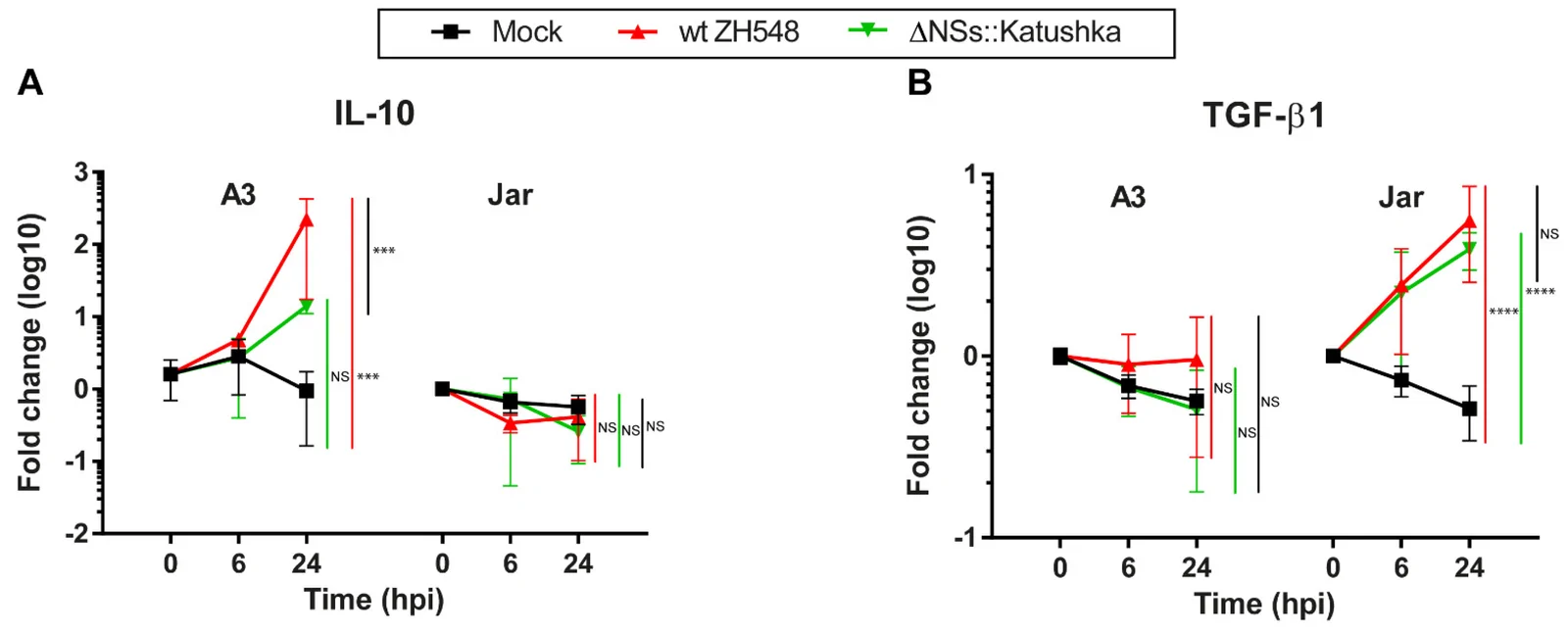
Differential messenger RNA (mRNA) expression of immunosuppressive/T regulatory cell (Treg) cytokines in A3 (immortalised human normal trophoblast) and Jar (human choriocarcinoma) cell lines infected with two different Rift Valley fever virus (RVFV) strains. Both cell lines were infected at a multiplicity of infection of 1 and cell lysates were harvested at 6 or 24 h post-infection (hpi). The total cellular RNA was extracted from harvested cell lysates and used for detection of interleukin (IL)-10 (**A**) and transforming growth factor (TGF)-β1 (**B**) mRNA by using reverse transcription-quantitative polymerase chain reaction (RT-qPCR) with TaqMan^®^ FAM/MGB probe assays. Experiments were performed in triplicate and repeated two times with similar results. The symbol plus error bar indicates the mean ± standard deviation. Statistical significance was determined by one-way analysis of variance (ANOVA) plus Dunnett’s post hoc analysis. The data from 24 hpi was used for multiple group comparison and the significance of *p* values is indicated by the asterisks; red line = mock vs. wt ZH548; green line = mock vs. ΔNSs::Katushka; black line = wt ZH548 vs. ΔNSs::Katushka (*** *p* < 0.001, **** *p* < 0.0001, NS = not significant).

We evaluated the immunosuppressive/Treg cytokine mRNA response to RVFV infection by analysing the expression of IL-10 and TGF-β1. Expression of IL-10 mRNA after RVFV infection was different for A3 and Jar cells. In the A3 cells at 24 hpi, the IL-10 mRNA expression was significantly upregulated (wt ZH548 = 223-fold), while ΔNSs::Katushka RVFV-infected A3 cells had no significant increase (Figure 6A).

By contrast, IL-10 mRNA expression was not upregulated in RVFV-infected Jar cells at 24 hpi (Figure 6A). For TGF-β1, neither RVFV variant affected the mRNA expression in infected A3 cells. On the other hand, TGF-β1 RNA expression was 4–5-fold upregulated in RVFV-infected Jar cells (Figure 6B).

In summary, RVFV infection evoked a differential mRNA expression of immunosuppressive/Treg cytokines in the two human trophoblast cell lines. IL-10 expression was induced in A3 cells by only the wt RVFV and TGF-β1 expression was induced in Jar cells by both variants (Figure 6).

### 3.7. Expression of Markers for the Cell Death Response and Cell Survival in Human Trophoblast Cell Lines after RVFV Infection

To identify alterations in the programmed cell death response and survival-related RNA expression in RVFV-infected human trophoblast cell lines, we determined mRNA expression of the apoptosis marker TP53, the autophagy marker MAP1LC3A, and the cell-survival marker NF-κB1.

**Figure 7:**
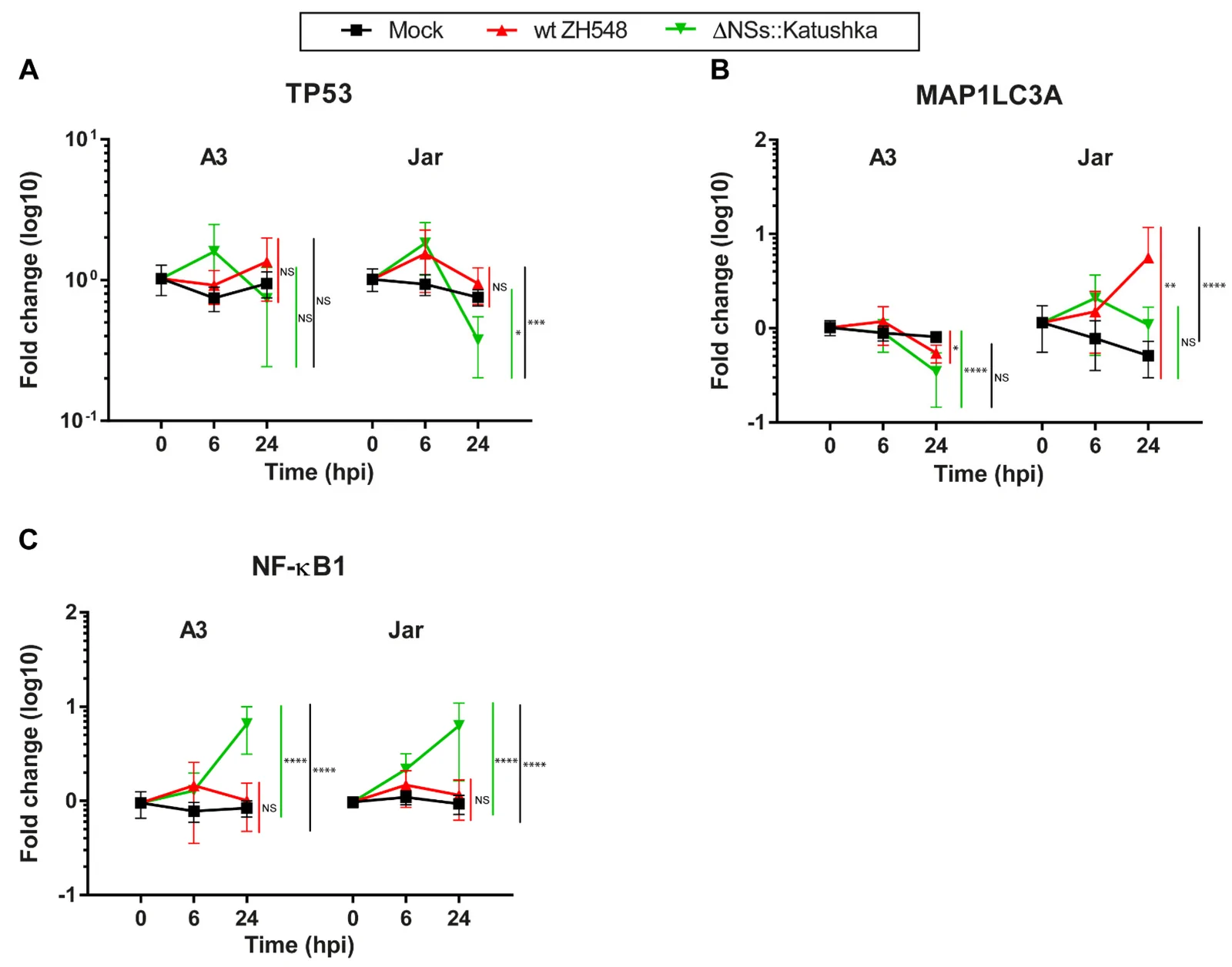
Programmed cell death and survival-related gene expression in in A3 (immortalised human normal trophoblast) and Jar (human choriocarcinoma) cell lines infected with two different Rift Valley fever virus (RVFV) strains. Both cell lines were infected at a multiplicity of infection of 1 and cell lysates were harvested at 6 or 24 h post-infection (hpi). The total cellular RNA was extracted from harvested cell lysate and used for detection of programmed cell death and survival-related gene expression of TP53 (**A**), MAP1LC3A (**B**), and NK-κB1 (**C**), by using reverse transcription-quantitative polymerase chain reaction (RT-qPCR) with TaqMan^®^ FAM/MGB probe assays. Experiments were performed in triplicate and repeated two times with similar results. The symbol plus error bar indicates the mean ± standard deviation. Statistical significance was determined by one-way analysis of variance (ANOVA) plus Dunnett’s post hoc analysis. The data from 24 hpi was used for multiple group comparison and the significance of *p* values is indicated by the asterisks; red line = mock vs. wt ZH548; green line = mock vs. ΔNSs::Katushka; black line = wt ZH548 vs. ΔNSs::Katushka (* *p* < 0.05, ** *p* < 0.01, *** *p* < 0.001, **** *p* < 0.0001, NS = not significant).

In general, RVFV infection did not induce alterations in TP53 transcription (Figure 7A). MAP1LC3A mRNA expression differed between A3 and Jar cells. At 24 hpi, MAP1LC3A RNA expression in A3 cells was not affected by infection with either RVFV variant, whereas in Jar cells, wt RVFV-infected cells showed a 5.6-fold increase in MAP1LC3A RNA expression (Figure 7B).

The pattern of NF-κB RNA expression during RVFV infection was similar in both cell lines; wt RVFV-infected cells showed minor changes in NF-κB1 RNA expression. By contrast, cells infected with ΔNSs::Katushka RVFV showed a significantly higher increase in NF-κB1 RNA expression at 24 hpi compared with wt RVFV (Figure 7C).

## 4. Discussion

In this study, we showed that two human trophoblast cell lines, one originating from normal trophoblasts and one from choriocarcinoma-transformed trophoblasts, were permissible to infection by two different variants of RVFV (wt and NSs deleted). These data suggest that villous trophoblasts might be a primary cell type for foetal RVFV infection, delineating a haematogenic vertical transmission pathway. It is probable that virus from infected trophoblasts covering the chorionic villi could reach the foetal blood in the foetal vessels located inside the chorionic villi and be further spread to the foetus, causing an intrauterine foetal infection. In immunocompetent Sprague Dawley rats, RVFV induced foetal demise through direct placental infection and RVFV could directly infect human placental chorionic villi isolated from mid-gestation placenta tissue [29].

The trophoblasts exhibited different cytokine profiles after infection. During wt RVFV infection, IFNα1 and λ mRNA expression was up-regulated and interestingly, IFNλ showed the highest increase in both trophoblast cell lines. Recently it was revealed that primary human trophoblasts constitutively release IFNλ1, and IFNλ1 has an important role in antiviral signaling at the maternal–fetal interface and protects syncytiotrophoblast from ZIKV infection [41]. Our results suggested that the trophoblast cells lines are not only prone to wt RVFV infection, but IFNλ1 is up-regulated as part of an antiviral defense against RVFV infection. wt RVFV infection did not show a general upregulation of inflammatory cytokines (IL-1β, IL-6, IL-8, and TNF-α), except for IL-1β and TNF-α in A3 cells.

IFNγ—which promotes a cytotoxic Th1 immune response—was, as expected, not expressed in the cell line developed from normal human villous trophoblasts, and only aberrantly expressed in the choriocarcinoma-transformed trophoblasts [40].

The NSs-deleted RVFV strain induced a stronger IFN and inflammatory response than the pathogenic wt RVFV. IFNλ showed the highest increase. The NSs of RVFV is a well-known IFN antagonist and it has been shown that RVFV NSs blocks IFN production by inhibiting host gene transcription and affects host RNA polymerase activities (e.g., TFIIH p62) [40,42]. Thus, as expected, infection with NSs-deleted RVFV resulted in higher expression of IFNβ1 and IFNλ mRNA than in the cells infected with wt ZH548. However, IFNα1 displayed a different pattern of mRNA expression: its mRNA was upregulated around 10-fold in all RVFV-infected cells, suggesting that RVFV NSs is not involved in the downregulation of IFNα1 mRNA in the two human trophoblast cell lines. NSs seems to mediate nuclear accumulation of mRNA following RVFV infection, although some mRNAs escape this block [43]. Thus, type I IFN mRNA expression during infection with the NSs-containing RVFV does not necessarily mean that cytokine proteins are produced.

Similarly to previously published work [32,34], the expression of the inflammatory cytokines (IL-1β, IL-6, IL-8, and TNF-α) after RVFV infection was upregulated to a greater extent in the absence of the RVFV NSs protein. All screened inflammatory cytokines showed a significant increase when the trophoblast cell lines were infected with NSs-deleted RVFV, and a significantly stronger induction than cells infected with wt RVFV. These data suggest that the posttranslational degradation of protein kinase R (PKR) by NSs protein has a role not only as an IFN antagonist, but may also affect the inflammatory cytokine responses directly or indirectly [42,44].

There were differences in cytokine induction between the two trophoblast cell lines. For example, IL-1β expression was upregulated in A3 cells but not in Jar cells; TNFα expression was upregulated for NSs-deleted RVFV infection in Jar cells but not in A3 cells; and IL-10 expression had a different induction/reduction profile depending on the cell line. One could speculate that even though A3 and Jar cells are both human trophoblast cell lines, the origin and the method of cell line establishment differed, and the immunological physiology and genetic background between these two cells are distinct [45]. The Jar cell line was derived from a choriocarcinoma, while the A3 cell line was derived from primary cells transformed in the laboratory.

During pregnancy, maternal immunity needs to fine-tune the balance between pro-inflammatory and anti-inflammatory immune responses, depending upon the stage of gestation, to enable both tolerance towards the foetus and defence against pathogens [20,46]. However, intrauterine infections and unnecessary inflammation could disrupt the balance in the maternal immune response and cause pregnancy complications [47]. Recent studies in mouse and human tissue explant models indicate that other emerging neurotropic viruses (Zika virus, West Nile fever virus, and Powassan virus) can infect the placenta and foetal brain, leading to foetal demise [48,49,50]. In these studies, the type I IFN signalling through the interferon-α/β receptor (IFNAR) within the foetus and foetal-derived placenta mediates severe complications, including foetal demise and severe growth restriction—for example, following Zika virus infection of pregnant mice [48,49,51].

In general, when a clinically detectable viral infection is present, elevated concentrations of cytokines, including IL-6, IL-1, IL-8, and TNF-α in amniotic fluid or cervicovaginal lavage of patients, is predictive of the onset of preterm labour [52,53]. Alongside previous studies, our finding indicated that RVFV infection in human trophoblast cell lines induced both IFN expression and transcription of inflammatory cytokines, which might be related closely to foetal demise and severe growth restriction [49,51,54,55,56]. Infection with NSs-deleted RVFV induced strong IFN transcription and inflammatory cytokine transcription, which might be associated with abortion and malformation. Similar findings were observed in a study using the RVFV Clone 13 strain (vaccine for domestic animals) in pregnant sheep [33]. In addition, we studied the mRNA expression of IL-10 and TGFβ1, immunosuppressive cytokines that promote adaptive and innate Treg cells, respectively. These cytokines are constitutively expressed at the foetal–maternal interface by a variety of cells including villous trophoblasts and are essential for maternal immune tolerance to the semi-allogeneic foetus. There was a differential expression of the immunosuppressive/Treg cytokines IL-10 and TGFβ1 in the infected trophoblast cell lines tested with both of the considered RVFV strains. For example, we observed that the immunosuppressive cytokine IL-10 was upregulated in A3 cells infected with wt RVFV, while A3 cells infected with NSs-deleted RVFV did not display any changes of IL-10. Further studies to understand the association between induced cytokines by RVFV infection leading to pregnancy pathologies is needed.

In this study, we chose to measure mRNA expression levels of cytokines instead of measuring biologically active molecules at the protein level. The main reason is that cytokines are small secreted peptides, sensitive to degradation if exposed to room temperature and freezing/thawing. As recently shown in cytokine analyses of cell culture supernatants, there is a risk for false negative results due to dilution of the cytokine proteins in the supernatant below the limit of detection of the analytical method used [57]. We used RT-qPCR because it is a highly specific, sensitive, and stable method; moreover, we have long-term experience with this method and recently tested its reliability for cytokine analyses. Considering the high sensitivity and reproducibility of the RT-qPCR method, our results suggest that determination of cytokine mRNA profiles could be used as a proxy for protein-mediated functions for specific purposes, such as comparisons between different experimental groups and in defining mechanistic pathways involved in the pathogenesis of various conditions [57].

In conclusion, we showed that two trophoblast cell lines were permissive for infection of two different variants of RVFV and we reported the cytokine mRNA profile after infection with these RVFV strains. As expected, wt RVFV with a functional NSs protein, displayed less increase of cytokine expression than the NSs-deleted RVFV strain. However, it was of interest to note the strong upregulation of IFNλ expression during wt RVFV infection. In comparison, the NSs-deleted RVFV strain showed upregulation of several cytokines, pointing to the potential for attenuated RVFV to cause a strong innate immune response.

In general, our findings highlight that RVFV infection caused dynamic gene expression changes, shown here by upregulation of mRNA for IFN types I–III and inflammatory cytokines, combined with a cell line-specific mRNA expression of TGFβ1 and IL-10, cytokines that are critical players in healthy pregnancy due to their ability to alter important biological functions such as immune suppression, cell–cell communication, tissue remodelling, and gene expression. This alteration in gene expression by RVFV infection has the potential to act as a teratogen and disrupt foetal and placental developmental pathways, leading to birth defects and other pregnancy complications such as miscarriage. Our study confirms and extends previous reports that the chorionic villi of human placenta are permissive to RVFV [12,29].

Furthermore, to our knowledge, this study is the first attempt to analyse the effect of RVFV infection on the cytokine mRNA profile in cells originating from human trophoblasts. Using immortalised cell lines for research is beneficial because they are cost-effective, easy to use, provide an unlimited supply of material, and bypass ethical concerns associated with the use of animal and human tissue [58]. However, major limitations are that they do not recapitulate the same phenotype or transcript profile as recently isolated primary cells. Therefore, they might not have the relevant attributes or functions of relatively normal cells [59]. In the near future, we plan to investigate other model systems (e.g., human placenta organoids and placenta explants) that better recapitulate the trophoblast phenotypes [60], to elucidate the altered gene expression in RVFV infection in human pregnancy. Such studies will provide important insights into the effect of RVFV on placental and foetal development and contribute to possible vaccine and drug development for the prevention and treatment of RVFV-associated pregnancy disorders.

## Data Availability

The data presented in this study are available on request from the corresponding author.

## Supplementary Materials

The following are available online at https://www.mdpi.com/article/10.3390/v13112265/s1, Figure S1: Standard curve of 18S ribosomal RNA control; Table S1: Average Ct values ± standard deviation for mRNA expression.
