# Supplementary material for: Rift Valley Fever Virus Propagates in Human Villous Trophoblast Cell Lines and Induces Cytokine mRNA Responses Known to Provoke Miscarriage
Source: Viruses. 2021 Nov 12;13(11):2265. doi: 10.3390/v13112265 (PMC8625252; doi:10.3390/v13112265)
Supplement: Supplementary file 1 [file viruses-13-02265-s001.zip › viruses-1452945-Supplementary.pdf]

viruses

MDPI

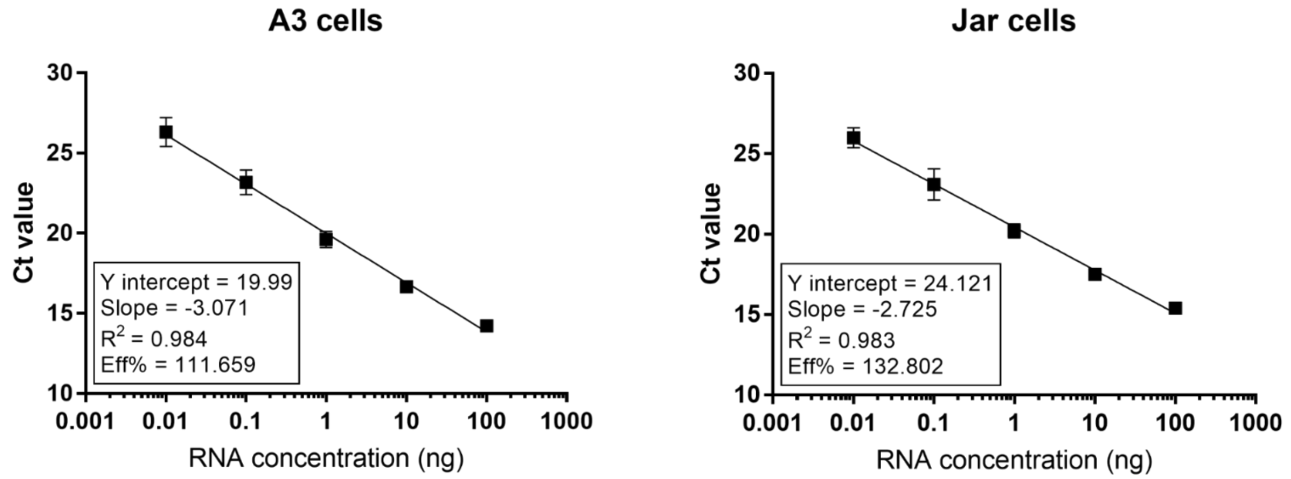

**18S ribosomal RNA control**	**Cell Line**	**RNA concentration (ng)**					
		**0.01**	**0.1**	**1**	**10**	**100**	**NTC**
average Ct values ± standard deviation	A3	26.31 ± 0.74	23.17 ± 0.63	19.60 ± 0.40	16.65 ± 0.11	14.22 ± 0.15	Undetermined
	JAR	25.98 ± 0.51	23.00 ± 0.79	20.21 ± 0.35	17.50 ± 0.17	15.40 ± 0.04	

Supplementary Figure 1. Standard Curve of 18S ribosomal RNA control (ThermoFisher, Cat No. 4319413E, VIC™/ MGB probe, primer limited). Each data point represents the average Ct values ± standard deviation (*n*=3). The dynamic range of total RNA extracted from A3, and Jar cells were used to generate a standard curve of r18S ribosomal RNA control. As a result, all initial template concentrations plotted on the graph appear to be in the linear dynamic range for the system.

Supplementary Table 1. Average Ct values ± standard deviation for mRNA expression. RVFV, cytokines, and the endogenous control gene 18S RNA, in A3 and Jar cells. Before and at different time points after RVFV infection (*n* = 6).

Cell Line	RVFV or Cytokines	Mock-infection			wt ZH548		ΔNSs:Katuskha		18s rRNA
		0h	6h	24h	6h	24h	6h	24h	
**A3**	**RVFV**	36.51 ± 1.33	38.07 ± 0.89	34.26 ± 2.04	23.58 ± 0.92	20.34 ± 0.37	24.58 ± 0.85	18.51 ± 0.24	16.46 ± 0.89
	**IFNα1**	27.72 ± 0.48	26.44 ± 0.26	26.21 ± 0.45	26.12 ± 0.31	25.63 ± 0.33	26.41 ± 0.54	25.82 ± 0.42	16.02 ± 0.67
	**IFNβ1**	27.44 ± 0.41	26.18 ± 0.39	26.12 ± 0.38	25.86 ± 0.51	25.20 ± 0.41	26.28 ± 0.28	20.78 ± 0.38	15.53 ± 0.76
	**IFNγ1**	Undetermined [1]	38.19 ± 1.70	39.17 ± 0.70	39.45 ± 0.00	39.65 ± 0.31	39.24 ± 0.10	39.24 ± 0.33	16.90 ± 0.75
	**IFNλ**	38.85 ± 0.58	39.21 ± 0.34	36.20 ± 1.11	34.84 ± 1.82	27.40 ± 1.94	31.91 ± 2.44	20.68 ± 0.59	17.30 ± 0.72
	**IL-4**	38.50 ± 0.02	37.05 ± 0.16	38.41 ± 0.31	38.64 ± 0.03	35.81 ± 0.26	37.92 ± 0.73	35.61 ± 0.77	17.26 ± 0.80
	**IL-5**	35.80 ± 0.33	35.95 ± 0.50	35.63 ± 0.47	35.64 ± 0.28	32.16 ± 0.14	35.96 ± 0.26	36.64 ± 0.13	16.04 ± 0.66
	**IL-1β**	25.93 ± 0.87	25.85 ± 1.19	25.01 ± 0.81	24.44 ± 0.91	22.96 ± 1.18	26.97 ± 1.15	21.50 ± 1.01	17.52 ± 1.19
	**IL-6**	28.46 ± 1.39	28.68 ± 1.42	28.23 ± 1.41	28.99 ± 2.03	26.72 ± 0.87	28.58 ± 0.92	21.47 ± 1.16	14.11 ± 2.77
	**IL-8**	24.40 ± 0.70	25.90 ± 0.58	24.46 ± 0.21	25.40 ± 1.71	22.71 ± 0.35	25.56 ± 0.56	17.13 ± 0.33	15.93 ± 1.00
	**TNF-α**	36.93 ± 0.53	37.93 ± 0.85	36.98 ± 0.81	37.85 ± 1.20	34.07 ± 2.49	37.90 ± 1.51	29.26 ± 1.71	16.45 ± 0.77
	**IL-10**	38.00 ± 0.72	36.53 ± 0.78	38.38 ± 0.88	36.98 ± 0.38	32.13 ± 1.08	37.82 ± 0.79	35.29 ± 1.19	16.65 ± 0.73
	**TGF-β1**	26.98 ± 1.53	26.76 ± 1.56	27.23 ± 1.80	27.71 ± 2.24	27.71 ± 2.19	27.71 ± 1.71	28.52 ± 2.00	19.01 ± 2.38
	**TP53**	27.83 ± 0.31	27.74 ± 0.25	27.46 ± 0.44	27.92 ± 0.56	28.07 ± 0.61	27.51 ± 0.59	29.18 ± 0.27	16.92 ± 0.77
	**LC3**	27.66 ± 1.15	27.35 ± 1.23	27.47 ± 1.52	28.23 ± 0.90	29.00 ± 0.61	28.07 ± 1.26	29.76 ± 1.21	19.96 ± 2.43
	**NF-κB**	32.03 ± 0.27	32.02 ± 0.40	31.86 ± 0.17	31.84 ± 0.60	32.72 ± 0.26	32.08 ± 0.28	30.26 ± 0.30	17.84 ± 0.97
**JAR**	**RVFV**	34.75 ± 2.01	35.72 ± 1.25	34.26 ± 1.86	25.03 ± 0.51	19.50 ± 3.17	20.62 ± 1.74	14.87 ± 1.46	16.57 ± 0.98
	**IFNα1**	28.14 ± 0.48	28.10 ± 0.31	28.78 ± 0.26	26.48 ± 0.19	26.04 ± 0.56	26.87 ± 0.38	26.35 ± 0.33	15.85 ± 0.84
	**IFNβ1**	27.37 ± 0.54	27.77 ± 0.54	28.74 ± 0.70	26.62 ± 0.49	26.26 ± 0.27	25.71 ± 0.44	21.22 ± 0.39	15.38 ± 0.87
	**IFNγ1**	36.44 ± 0.42	37.22 ± 0.86	36.83 ± 1.28	37.20 ± 0.60	37.21 ± 1.27	36.65 ± 1.19	35.32 ± 0.40	16.97 ± 0.83
	**IFNλ**	35.91 ± 0.97	36.00 ± 1.23	35.61 ± 1.10	29.85 ± 2.64	27.34 ± 3.80	28.49 ± 3.20	19.45 ± 0.18	17.14 ± 0.80
	**IL-4**	Undetermined	Undetermined	Undetermined	38.24 ± 0.00	38.01 ± 0.00	Undetermined	Undetermined	17.14 ± 0.97
	**IL-5**	33.88 ± 0.33	33.56 ± 0.01	33.65 ± 0.25	34.59 ± 0.10	34.39 ± 0.46	34.52 ± 0.22	33.22 ± 0.25	15.96 ± 1.06
	**IL-1β**	36.11 ± 0.56	Undetermined	37.09 ± 1.08	35.21 ± 0.64	35.25 ± 1.08	34.77 ± 0.93	32.41 ± 0.32	17.34 ± 1.11
	**IL-6**	32.32 ± 0.95	31.40 ± 1.09	30.78 ± 1.13	31.01 ± 1.44	32.07 ± 0.87	30.16 ± 0.98	25.47 ± 0.99	13.90 ± 2.87
	**IL-8**	34.79 ± 1.20	35.30 ± 1.46	35.66 ± 1.02	34.16 ± 1.82	34.85 ± 0.92	33.67 ± 1.60	24.40 ± 1.20	16.06 ± 0.97
	**TNF-α**	34.28 ± 1.52	34.40 ± 2.21	33.07 ± 1.89	33.97 ± 2.57	30.47 ± 4.38	31.00 ± 3.05	24.66 ± 1.97	16.47 ± 0.88
	**IL-10**	33.80 ± 2.00	34.25 ± 2.27	33.81 ± 2.17	35.98 ± 1.50	36.88 ± 2.02	35.57 ± 2.89	36.56 ± 0.40	16.57 ± 0.80
	**TGF-β1**	29.81 ± 2.63	30.68 ± 2.83	30.41 ± 2.09	29.91 ± 3.11	28.68 ± 2.80	30.09 ± 3.14	29.00 ± 2.18	19.02 ± 2.39
	**TP53**	28.26 ± 0.20	28.11 ± 0.12	28.45 ± 0.36	28.69 ± 0.94	29.10 ± 0.97	28.51 ± 0.97	30.27 ± 0.24	16.72 ± 0.99
	**LC3**	36.27 ± 1.06	36.49 ± 1.16	36.13 ± 0.82	36.41 ± 0.86	36.35 ± 1.98	35.94 ± 0.94	36.71 ± 0.46	19.80 ± 2.42
	**NF-κB**	32.31 ± 0.16	31.93 ± 0.24	32.00 ± 0.28	32.37 ± 0.19	33.42 ± 0.51	31.83 ± 0.15	30.80 ± 0.55	17.71 ± 0.88

[1] Undetermined Ct values were excluded from this study.
